# Survey of intention among public health nurses in providing solution‐focused parenting support

**DOI:** 10.1111/phn.13032

**Published:** 2021-12-13

**Authors:** Liesbeth Theuns‐Boumans, Jolanda Mathijssen, Carin Rots‐de Vries, Ien van de Goor

**Affiliations:** ^1^ Department Tranzo Academic Collaborative Centre Youth, Tilburg University Tilburg The Netherlands; ^2^ Avans University of Applied Sciences Bachelor of Nursing Breda The Netherlands; ^3^ Department Tranzo Tilburg University Academic Collaborative Centre Public Health Tilburg The Netherlands

**Keywords:** community health nursing, intention, parenting support, public health nursing practice, solution‐focused, Theory of Planned Behavior

## Abstract

**Objective:**

Parenting support has shifted from a problem‐focused to a strengths‐based solution‐focused approach. This study surveyed public health nurses to explain their intention to provide solution‐focused parenting support in their practice.

**Design:**

The design of this study was cross‐sectional.

**Sample:**

The initial sample included 781 public health nurses who were employed with various youth healthcare organizations in the Netherlands.

**Measurements:**

Based on the Theory of Planned Behavior, a questionnaire was developed and administered to measure (a) behavioral, normative, and control beliefs, (b) attitudes, subjective norm, and perceived behavioral control, and (c) intention. The data were subjected to structural equation modeling.

**Results:**

A total of 449 (57.5%) public health nurses completed questionnaires. Associations as indicated by the Theory of Planned Behavior were confirmed with the exception of that between perceived behavioral control and intention. Statistically significant paths and correlations were added. The final model accounted for 53% of the variance in the intention to perform solution‐focused parenting support.

**Conclusions:**

In this study, public health nurses strongly intended to provide solution‐focused parenting support, thus indicating their acceptance of the approach. Their intention was predominantly associated with subjective norm.

## 1 BACKGROUND

In recent years, the guidelines and interventions for parenting support have shifted from a problem‐focused approach to a solution‐focused approach (Carr et al., 2017; Polaschek & Polaschek, 2007; Wells et al., 2014). More specifically, solution‐focused parenting support is a strengths‐based approach in which positive, future‐oriented goals are established. In this context, practitioners must assess parents to determine their current strengths and available resources, thus identifying the first steps parents should take in moving toward their objectives. The parental perspective is positioned at the center during each stage of this support process, with professionals leading the parents from behind (De Jong & Berg, 2013; De Shazer et al., 1986). Meanwhile, scholarly evidence continues to show the promising effects of this approach (Bond et al., 2013; Gingerich & Peterson, 2013; Stams et al., 2006). When compared to problem‐focused parenting support, the solution‐focused approach adds value through its specific focus on sustainably increasing motivation, self‐confidence, and problem‐solving capacity among the parents (De Jong & Berg, 2013).

In healthcare practice, it takes a complex process to actually change and sustainably implement new guidelines (Grol & Buchan, 2006; Grol et al., 2013). Previous investigators have described this as “a planned process and systematic introduction of innovations and/or changes of proven value; the aim being that these are given a structural place in professional practice, in the functioning of organizations or in the healthcare structure” (Zorg Onderzoek Nederland, 1997). According to Grol et al. (2013), the implementation process can be divided into the five following stages:

- Orientation—the target group develops awareness, involvement, and interest in the presented topic.
- Insight—the target group gains a factual understanding of the innovation, thus raising their insights into their own practices.
- Acceptance—focus is placed on the development of positive attitudes, the motivation for change, and the positive intention to change.
- Change—an actual shift occurs in practice; meanwhile the benefits and values of the innovation are validated.
- Maintenance – the target group integrates new routines into daily practice and organizes financial and structural support.

In 2014, the youth healthcare (YH) initiated the implementation of solution‐focused parenting support in the Netherlands, with relevant activities rooted in the orientation and insight stages. At that time, organizational policies and parenting support guidelines were altered to reflect the new approach. Through various channels, public health nurses (PHNs) were informed about the intended shift in parenting support to facilitate their orientation to the subject, and the content of the solution‐focused guideline of parenting support was implemented through various activities (e.g., informative team sessions, training in solution‐focused parenting support, and participation in peer supervision sessions). In this regard, PHNs are now expected to have progressed through stage two of implementation (insight). As a result, they are supposed to accept solution‐focused parenting support as a valuable new approach, which in turn is supposed to change their actual support behavior (Grol et al., 2013). However, it is currently unclear whether these PHNs have truly accepted solution‐focused parenting support.

One way to determine if the stage of acceptance of solution‐focused parenting support was established is to examine PHNs’ intention to provide it in practice. Indeed, Grol and Wensing (2004), and Grol et al. (2013) assert that a key element of the acceptance stage is the intention to actually change one's behavior. Here, intention can be defined as “how hard people are willing to try, or how much of an effort they are planning to exert, in order to perform the behavior” (Ajzen, 1991, p. 181).

**FIGURE 1:**
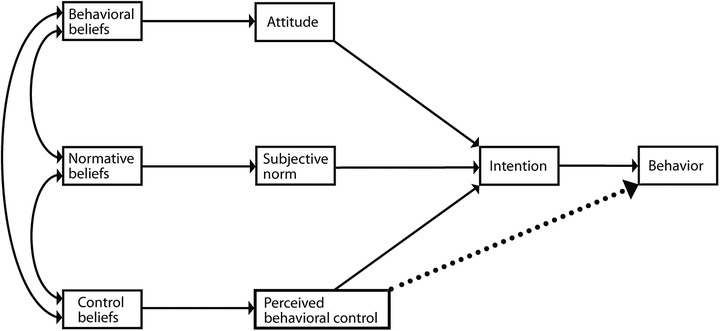
Theory of Planned Behavior (Ajzen, 1991, 2002)

As shown in Figure 1, the Theory of Planned Behavior (TPB) posits that intention is the most proximal antecedent of behavior and behavioral change; it specifically indicates an individual's intention for a given behavior based on their attitude, subjective norm, and perceived behavioral control (PBC) (Ajzen, 1991). Attitude refers to the positive or negative evaluation of a given behavior by a specific individual, while subjective norm refers to experienced social pressures to perform the behavior of interest, and PBC includes both the concept of capability and controllability, referring to whether an individual views a given behavior as easy or difficult to perform. In the theoretical context, PBC is related to both intention and behavior (Ajzen, 1991).

TPB entails that the respective antecedents of attitude, subjective norm, and PBC are behavioral, normative, and control beliefs. Behavioral beliefs refer to an individual's expectations or the subjective probability that performing a given behavior will lead to a certain outcome, while normative beliefs refer to the expected approval (or disapproval) by significant others to provide a given behavior, and control beliefs refer to the subjective probability of the presence of specific impeding and facilitating factors to perform the given behavior (Ajzen, 1991, 2002).

Previous studies have shown that TPB is useful for predicting and explaining the intention of nurses in various areas of their professional behavior (Godin et al., 2008). For example, TPB concepts have explained 34% of the intention to use clinical guidelines in the hospital setting (Kortteisto et al., 2010), 50% of the intention to use filter needles when preparing parenteral medications (Gagnon et al., 2015), and 56% of the intention to adhere to hand hygiene guidelines in intensive care and post‐intensive care units (O'Boyle et al., 2001). These nursing behaviors are more medically oriented, while solution‐focused parenting support behaviors are more socially oriented. Nevertheless, the use of clinical guidelines in the hospital can be viewed as a more general nursing behavior that is comparable to solution‐focused parenting support behaviors, albeit in a different setting. As such, the explained variance of the intention to provide solution‐focused parenting support is expected to be at least 34% among PHNs.

There is currently insufficient evidence on whether PHNs in the Netherlands actually intend to provide solution‐focused parenting support. This knowledge is important for determining whether they have progressed through the acceptance stage in regard to implementing this support approach. As such, this study used the TPB framework to explore and explain the intention of PHNs in the Netherlands to provide solution‐focused parenting support. We developed the following research question: To what extent can the intention of PHNs to provide solution‐focused parenting support be explained via the TPB concepts of attitude, subjective norm, and PBC?

## 2 METHODS

### 2.1 Design

The design of this study was cross‐sectional. An internet‐based survey was developed and administered among a sample of PHNs employed at YH organizations throughout the Netherlands between November 2017 and May 2018.

### 2.2 Setting and sample

YH is a regionally organized Dutch preventive public health service for all children aged 0–18 and their families. It is free of charge. Its contents are defined in the Dutch Public Health Act (Ministry of Health, Welfare and Sports, 2008). The main goal is to monitor and promote the health and wellbeing of all children living in the Netherlands.

During different standardized consultations, medical doctors and PHNs screen and monitor the physical, psychosocial, and cognitive health of children; for example, assessments include nutrition and weight, motor development, language development, and behavioral competence. These professionals also provide parents and their children with health education to promote healthy child development. If risks or mild problems are detected (e.g., developmental gaps or behavioral problems), then YH professionals can intervene (i.e., early intervention). If more severe problems are noticed, then YH professionals can direct the parents and their children to more specialized professionals such as pediatricians, psychologists, or physiotherapists. In cases of mild parenting problems, PHNs provide parenting support as an early intervention.

The 48 regional YH organizations employ approximately 3000 PHNs (Jambroes et al., 2015). In this study, 20 of those organizations were randomly selected and asked to recruit participants among PHNs from their respective staffs. However, four organizations did not react despite multiple requests, while four others declined due to heavy workloads. Ultimately, 12 organizations agreed to participate, resulting in a final sample of 781 PHNs. All provided their consent to participate.

### 2.3 Measures

#### 2.3.1 Instrument

Francis et al. (2004) composed guidelines for the development of a TPB questionnaire. In this study, we used those guidelines to develop the Intention to Provide Solution‐Focused Parenting Support questionnaire (I‐SFPS), which includes scales for (a) behavioral, normative, and control beliefs; (b) attitude, subjective norm, and PBC; and (c) intention. In a previous study, we analyzed the most salient behavioral, normative, and control beliefs of PHNs using a factor analysis procedure, then measured the internal consistency of the obtained factors. That analysis revealed three internal consistent beliefs scales that equated to the behavioral (10 items; α = .79), normative (four items; α = .80) and control beliefs (eight items; α = .64) from the TPB [in review].

The first draft of the I‐SFPS was developed using the Qualtrics^®^ XM software (2019), and included an introductory text with an informed consent procedure. It included 37 measures related to the TPB constructs, with items answered on a 7‐point Likert scale (1 = totally disagree/difficult; 7 = totally agree/easy), as recommended in TPB research (Ajzen, 1991; Francis et al., 2004). We tested the clarity, comprehensibility, size, convenience, and acceptability of the I‐SFPS. Interviews with five PHNs were accomplished to document their opinions. Following this advice, the introductory text was adapted and the word order was changed for two questions to improve clarity. The I‐SFPS can be viewed in the additional file enclosed with this manuscript (Appendix A. Intention to Provide Solution‐Focused Parenting Support questionnaire).

### 2.4 Procedures

#### 2.4.1 Data collection

The sample of PHNs was invited through emails that also contained links to the digital I‐SFPS, and informed consent procedure. They were informed of the study aim, data anonymization, data storage procedure, voluntary nature of participation, and possibility to withdraw at any time. Three reminders were sent over the six‐week collection period. All study methods were approved by the Ethics Review Board of Tilburg University.

#### 2.4.2 Data analysis

First, missing items were dealt with by either excluding the case from further analysis (*n* = 12) or via mean substitution (*n *= 4) (Tabachnick & Fidell, 2007). Second, internal consistency and reliability (Cronbach's alpha) were individually determined for attitude (four items; α = .93), subjective norm (three items; α = .80), PBC (two items; α = .86), and intention (three items; α = .87). Items were deleted if they significantly inflated the internal consistency and reliability (items C10, C12, and C15 were deleted). Third, since the participants (level 1) were nested within their organizations (level 2), the data were hierarchically organized. We calculated the intraclass correlation coefficient (ICC) to test whether it was necessary to control for the organizational effect (Shek & Ma, 2011). The ICC (proportion of total variation in variables, which is attributable to differences between organizations) indicates that multilevel modeling is necessary if the proportion of explained variance is 25% or higher (Heinrich & Lynn, 2001). In this study, the ICCs of the variable scales ranged between 0.5% and 4.5%, in which case multilevel modeling was not needed. Lastly, we used IBM SPSS AMOS (version 24.0) to conduct structural equation modeling (SEM). The variable scales were included as observed variables, while the TPB model (Figure 1) was estimated, evaluated, and improved through a modification approach toward a theoretical defensible fit of the model to the data. Improvements in model fit were evaluated using the Chi‐square difference test (Tabachnick & Fidell, 2007), with final model fit determined based on both the Chi‐square statistic (*p* > .05 indicating good model fit) and RMSEA test statistic (*p* < .06 indicating good model fit) (Schreiber et al., 2006; Ullman & Bentler, 2013).

## 3 RESULTS

This study surveyed the TPB concepts to explain the intention among PHNs to provide solution‐focused parenting support. A total of 449 participants (57.5%) completed the I‐SFPS questionnaire. All 12 organizations were represented in this sample, with response percentages at each organization reaching at least 43.6%. Table 1 shows the participant characteristics.

**TABLE 1:** Characteristics of the surveyed public health nurses

Characteristics	*N* (449)
Gender	
Female	439 (97.8%)
Male	10 (2.2%)
Age (years)	
20−30	64 (14.2%)
31−40	93 (20.7%)
41−50	97 (21.6%)
51−60	148 (33%)
61−67	47 (10.5%)
Work experience (years)	
0−5	98 (21.8%)
6−10	65 (14.5%)
11−15	64 (14.3%)
16−20	81 (18%)
21−25	34 (7.6%)
26−30	55 (12.2%)
31−35	34 (7.6%)
36−40	14 (3.1%)
>40	4 (0.9%)

Table 2 shows the means (M) and standard deviations (SD) for all variables and their correlations. Overall, the participants returned positive scores for the TPB variables. Further, all variables were correlated. On a scale ranging from 1 to 7, the mean intention to provide solution‐focused parenting support was 6 (SD = .92).

**TABLE 2:** Descriptions of variables used in the model

	Intention	Attitude	Subjective norm	PBC	Normative beliefs	Behavioral beliefs	Control beliefs
Intention (M = 6.0; SD = .92)	1						
Attitude (M = 6.11; SD = .85)	.587^**^	1					
Subjective norm (M = 5.42; SD = 1.06)	.652^**^	.459^**^	1				
PBC (M = 5.45; SD = 1.05)	.350^**^	.410^**^	.309^**^	1			
Normative beliefs (M = 4.92; SD = 1.08)	.461^**^	.367^**^	.654^**^	.246^**^	1		
Behavioral beliefs (M = 5.54; SD = .65)	.475^**^	.624^**^	.347^**^	.478^**^	.296^**^	1	
Control beliefs (M = 4.24; SD = .84)	.221^**^	.269^**^	.218^**^	.575^**^	.277^**^	.391^**^	1

** Correlation is significant at the 0.01 level (2‐tailed); PBC = perceived behavioral control.

**FIGURE 2:**
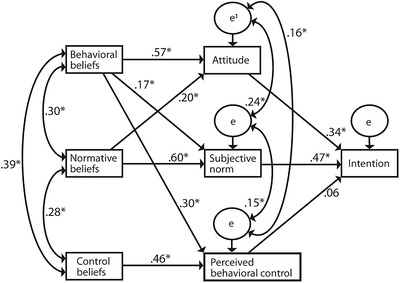
Final model explaining the intention of participants to provide solution‐focused parenting support. *statistically significant at *p* < .01 level. ¹error term/residual variable

Model fit was achieved in six steps (Table 3). Each modification step improved the model significantly. The final model (model 7; Figure 2) had a non‐significant Chi‐square statistic and RMSEA < .05, both of which indicated a good model fit (Schreiber et al., 2006).

**TABLE 3:** Model fit modifications

						Differences between models	
Model	Modification	Chi square	df	P‐value	RMSEA	Chi‐square (df)	*p*‐value
1		155.378	12	.000	.163		
2	NB → A	127.741	11	.000	.154	27.637 (1)	<.05
3	BB → PBC	74.243	10	.000	.120	53.498 (1)	<.05
4	eSN ↔ eA	48.626	9	.000	.099	25.617 (1)	<.05
5	BB → SN	27.978	8	.000	.075	20.648 (1)	<.05
6	eSN ↔ ePBC	21.502	7	.003	.068	6.476 (1)	<.05
7	eA ↔ ePBC	9.558	6	.145	.036	11.944 (1)	<.05

Abbreviations: A, Attitude; BB, behavioral beliefs; e, error term; NB, normative beliefs; SN, subjective norm.

The analysis revealed statistically significant associations between behavioral beliefs and attitude, between normative beliefs and subjective norm, and between control beliefs and PBC. Moreover, associations between attitude and intention, and between subjective norm and intention were found. Furthermore, statistically significant correlations were found between behavioral beliefs and normative beliefs, between behavioral beliefs and control beliefs, and between normative beliefs and control beliefs. The association between PBC and intention was non‐significant.

Statistically significant paths were also added to the original paths in the TPB. These paths connected behavioral beliefs to subjective norm, behavioral beliefs to PBC, and normative beliefs to attitude. Finally, correlations were added between the error terms of attitude and subjective norm, the error terms of subjective norm and PBC, and the error terms of attitude and PBC. Together, this final model accounted for 53% of the variance in the intention to perform solution‐focused parenting support.

## 4 DISCUSSION

While the solution‐focused approach to parenting support is now the preferred method, it is unclear whether PHNs intend to provide solution‐focused parenting support into their professional practice. This study investigated to what extent the intention of PHNs to provide solution‐focused parenting support could be explained via the TPB concepts of attitude, subjective norm, and PBC. The findings demonstrate that the TPB concepts subjective norm and attitude explained the intention of PHNs to provide solution‐focused parenting support.

Overall, the results showed a strong intention to provide solution‐focused parenting support. Based on the TPB concepts of attitude, subjective norm, and PBC, the explained variance of this intention was high when compared to the general intention to follow clinical guidelines (Kortteisto et al., 2010). The explained variance of intention was congruent with previous studies that examined more specific nursing behaviors, such as the adherence to hand hygiene protocols and the use of filter needles when preparing medications for parenteral injections (Gagnon et al., 2015; O'Boyle et al., 2001). However, such comparisons are limited due to differences in the target behaviors and nursing contexts. Other studies among nurses have reported explained variances in behavioral intention up to 70% (Côté et al., 2012; Godin et al., 2008; Lee & Kang, 2020). However, comparison of results is not possible since these studies included other predicting concepts in addition to the TPB model (e.g., moral norm and past behaviors) whereas our study did not.

Of particular note, the model used in this study showed strong effects for behavioral beliefs, which were positively related to attitude, subjective norm, and PBC. While TPB entails a strong association between behavioral beliefs and attitude, the associations between behavioral beliefs and both subjective norm/PBC were surprising. Given that this study employed a cross‐sectional design, it may be hypothesized that behavioral beliefs changed due to perceived subjective norm and PBC among the participants. For example, they may have perceived solution‐focused parenting support behaviors as socially desirable following the organizational implementation of related policies and guidelines. This may imply that participants experienced social pressure, thus influencing their view that solution‐focused parenting support was both positive and beneficial. In this regard, behavioral beliefs may change due to experiences in which strong subjective norm is applied. Further, PHNs who perceive strong behavioral control in the context of performing solution‐focused parenting support may also be more likely to think positively about the associated benefits and characteristics. However, these hypotheses contrast with the original directions of the associations as modeled via TPB (Figure 1). Additional research is therefore needed to clarify any such relationships.

We also found that subjective norm was most strongly associated with the intention to provide solution‐focused parenting support, thus supporting previous studies showing this association among other nurses (Kortteisto et al., 2010; O'Boyle et al., 2001). Meanwhile, attitude was also positively related to the intention to provide solution‐focused parenting support, which similarly supports previous research showing the same type of association for various nursing behaviors under the TPB framework (Gagnon et al., 2015; Kortteisto et al., 2010; Lee & Kang, 2020). Thus, the current results imply that evaluating the benefits of solution‐focused parenting support and/or being influenced by positive subjective norms to provide this approach are associated with PHNs strong intentions to provide this support.

For YH organizations in the Netherlands, the implementation of solution‐focused parenting support can be enhanced in two ways. The first involves persuasive interventions aimed at increasing positive attitudes about the method among PHNs, while the second involves interventions aimed at increasing the experienced positive norm exhibited by colleagues. Since attitude and subjective norm are correlated, these interventions should have complementary effects. Following said interventions, longitudinal research will be needed to measure the actual effects at different timepoints (i.e., at baseline, during, and after).

In this study, PBC was not related to intention, which is remarkable. Indeed, this finding contrasts with previous studies (Gagnon et al., 2015; Kortteisto et al., 2010). This may be because PBC in the I‐SFPS was restricted to measuring perceived capability. According to TPB, the PBC construct is comprised of two components, including perceived capability and actual control. In this study, the actual control component of PBC was excluded from analysis due to internal consistency and reliability issues. It is therefore difficult to compare the current results with the outcomes of studies that included the actual control component of PBC. Further, the lack of a statistically significant association between PBC and intention in this study may be explained due to the fact that our analysis controlled for correlations between both PBC and attitude (0.41) and PBC and subjective norm (0.31) when calculating the unique contribution of PBC to the explained variance of intention (Tabachnick & Fidell, 2007). Additional research is needed to further develop and test the PBC construct in the I‐SFPS and assess the associations between PBC and intention.

This study had several strengths. First, there were 449 participants, which is generally accepted as sufficient for obtaining reliable results from SEM (Schumacker & Lomax, 2004). Moreover, the rigorous development and testing of the I‐SFPS and adequate handling of missing data increased the internal validity of the results. On the other hand, there were also some limitations. One was the cross‐sectional design, which confines conclusions about the direction of the relationships in the model. In future research, a longitudinal design should be implemented to provide more detailed insights into the associations between TPB concepts. In addition, participants with the tendency to give more socially desirable answers may have positively biased the results. This also highlights the need to consider the self‐reported nature of the questionnaire data when making interpretations. Finally, we did not ask participants for their specific background characteristics. Despite the large sample size, this further limits generalizability.

In sum, the PHNs in this study expressed a strong intention to provide solution‐focused parenting support, thus indicating their readiness for change. This is promising, since such a strong intention should help ensure the successful and sustainable implementation of solution‐focused parenting support among PHNs employed with YH organizations. Moreover, the intention to provide the method was predominantly determined by perceived subjective norm. As such, future implementation activities should be geared toward increasing perceived positive subjective norm. One way to accomplish this is to solicit the help of close colleagues during implementation activities. Meanwhile, we did not measure actual parenting support behaviors. For that reason, behavioral intention remained a mental state in the context of this study, and was thus an uncertain predictor of actual behavior. Here, a strong intention does not necessarily indicate that support behaviors had already been altered toward the solution‐focused approach in daily practice. According to Grol et al. (2013), behavioral intention indicates the acceptance of an innovative intervention. The next step should be to increase actual behaviors aimed at solution‐focused parenting support; for example, PHNs can be included in participatory action research as part of their daily practice, with a focus on detecting what works for them when changing toward solution‐focused parenting support, and by simultaneously changing their practice in compliance with this support approach (Cooperrider et al., 2008; Cusack et al., 2018; Kemmis et al., 2014).

## Data Availability

The data that support the findings of this study are available on request from the corresponding author. The data are not publicly available due to privacy or ethical restrictions.

## APPENDIX A INTENTION TO PROVIDE SOLUTION‐FOCUSED PARENTING SUPPORT QUESTIONNAIRE (I‐SFPS)

### A.1

**Table:**

Demographic questions		
A1.	What is your gender?	Female
		Male
A2.	What is your age?	20−30
		31−40
		41−50
		51−60
		61‐67
A3.	At which organization are you employed?	▼
A4.	How many years have you been working as a public health nurse?	
		0−5
		6−10
		11−15
		16−20
		21−25
		26−30
		31−35
		36‐40
		>40
Behavioral beliefs questions		
If I provide or would provide solution‐focused parenting support to parents with mild parenting problems …		
B1	… the same result is achieved in less time.	Totally disagree 1‐2‐3‐4‐5‐6‐7 Totally agree
B2	… the motivation of parents is stimulated.	Totally disagree 1‐2‐3‐4‐5‐6‐7 Totally agree
B3	… it stimulates a good contact with parents.	Totally disagree 1‐2‐3‐4‐5‐6‐7 Totally agree
B4	… the parents’ perspective is central in the support process.	Totally disagree 1‐2‐3‐4‐5‐6‐7 Totally agree
B5	… I adopt a positive approach.	Totally disagree 1‐2‐3‐4‐5‐6‐7 Totally agree
B6	… the support process is less time‐efficient than other support approaches.	Totally disagree 1‐2‐3‐4‐5‐6‐7 Totally agree
B7	… parents become more active in finding a suitable solution.	Totally disagree 1‐2‐3‐4‐5‐6‐7 Totally agree
B8	… I cannot deploy my professional knowledge.	Totally disagree 1‐2‐3‐4‐5‐6‐7 Totally agree
B9	… the strengths of parents are being confirmed.	Totally disagree 1‐2‐3‐4‐5‐6‐7 Totally agree
B10	Solution‐focused parenting support is suitable in all cases of parenting support.	Totally disagree 1‐2‐3‐4‐5‐6‐7 Totally agree
Normative beliefs questions		
B11	Stakeholders think I should provide solution‐focused parenting support to parents with mild parenting problems.	Totally disagree 1‐2‐3‐4‐5‐6‐7 Totally agree
B12	The management of my organization wants me to provide solution‐focused parenting support to parents with mild parenting problems.	Totally disagree 1‐2‐3‐4‐5‐6‐7 Totally agree
B13	I assume my colleagues think I should provide solution‐focused parenting support to parents with mild parenting problems.	Totally disagree 1‐2‐3‐4‐5‐6‐7 Totally agree
B14	Nurseries, preschools, and schools expect me to provide solution‐focused parenting support to parents with mild parenting problems.	Totally disagree 1‐2‐3‐4‐5‐6‐7 Totally agree
Control beliefs questions		
B15	If I provide or would provide solution‐focused parenting support to parents with mild parenting problems, I work according to a concrete methodology.	Totally disagree 1‐2‐3‐4‐5‐6‐7 Totally agree

(Continues)

### APPENDIX A (Continued)

**Table:**

B16	If I provide or would provide solution‐focused parenting support to parents with mild parenting problems, I find it difficult to ask correct solution‐focused questions.	Totally disagree 1‐2‐3‐4‐5‐6‐7 Totally agree
B17	The time available in youth healthcare to provide solution‐focused parenting support to parents with mild parenting problems is insufficient.	Totally disagree 1‐2‐3‐4‐5‐6‐7 Totally agree
B18	The training “solution‐focused parenting support” as offered by the youth healthcare is sufficient.	Totally disagree 1‐2‐3‐4‐5‐6‐7 Totally agree
B19	For me, carrying out peer supervision sessions into solution‐focused parenting support is …	Difficult 1‐2‐3‐4‐5‐6‐7 Easy
B20	For me, providing solution‐focused parenting support to unmotivated parents is …	Difficult 1‐2‐3‐4‐5‐6‐7 Easy
B21	For me, gaining experience in solution‐focused parenting support in daily practice is …	Difficult 1‐2‐3‐4‐5‐6‐7 Easy
B22	I have insufficient knowledge about solution‐focused parenting support to parents with mild parenting problems.	Totally disagree 1‐2‐3‐4‐5‐6‐7 Totally agree
Intention questions		
C1	Generally, I expect to provide solution‐focused parenting support to parents with mild parenting problems	Totally disagree 1‐2‐3‐4‐5‐6‐7 Totally agree
C2	Generally, I intend to provide solution‐focused parenting support to parents with mild parenting problems	Totally disagree 1‐2‐3‐4‐5‐6‐7 Totally agree
C3	Generally, I want to provide solution‐focused parenting support to parents with mild parenting problems	Totally disagree 1‐2‐3‐4‐5‐6‐7 Totally agree
Attitude questions		
C4	Generally, I think providing solution‐focused parenting support to parents with mild parenting problems is	Bad 1‐2‐3‐4‐5‐6‐7 Good
C5	Generally, I think providing solution‐focused parenting support to parents with mild parenting problems is	Not helping 1‐2‐3‐4‐5‐6‐7 Helping
C6	Generally, I think providing solution‐focused parenting support to parents with mild parenting problems is	Unpleasant 1‐2‐3‐4‐5‐6‐7 Pleasant for me
C7	Generally, I think providing solution‐focused parenting support to parents with mild parenting problems is	Unimportant 1‐2‐3‐4‐5‐6‐7 Important
Subjective norm questions		
C8	Generally, it is expected of me that I provide solution‐focused parenting support to parents with mild parenting problems	Totally disagree 1‐2‐3‐4‐5‐6‐7 Totally agree
C9	People who are important to me at work think I should provide solution‐focused parenting support to parents with mild parenting problems	Totally disagree 1‐2‐3‐4‐5‐6‐7 Totally agree
C10	Generally, I feel under social pressure to provide solution‐focused parenting support to parents with mild parenting problems	Totally disagree 1‐2‐3‐4‐5‐6‐7 Totally agree
C11	People who are important to me at work want me to provide solution‐focused parenting support to parents with mild parenting problems	Totally disagree 1‐2‐3‐4‐5‐6‐7 Totally agree
Perceived behavioral control questions		
C12	Whether I provide solution‐focused parenting support to parents with mild parenting problems is entirely up to me	Totally disagree 1‐2‐3‐4‐5‐6‐7 Totally agree
C13	For me, providing solution‐focused parenting support to parents with mild parenting problems is	Difficult 1‐2‐3‐4‐5‐6‐7 Easy
C14	I am confident that I could provide solution‐focused parenting support to parents with mild parenting problems if I wanted to	Totally disagree 1‐2‐3‐4‐5‐6‐7 Totally agree
C15	The decision to provide solution‐focused parenting support to parents with mild parenting problems is beyond my control	Totally disagree 1‐2‐3‐4‐5‐6‐7 Totally agree
